# Socioeconomic Factors Associated With Glycemic Measurement and Poor HbA1c Control in People With Type 2 Diabetes: The Global DISCOVER Study

**DOI:** 10.3389/fendo.2022.831676

**Published:** 2022-04-22

**Authors:** Marília B. Gomes, Fengming Tang, Hungta Chen, Javier Cid-Ruzafa, Peter Fenici, Kamlesh Khunti, Wolfgang Rathmann, Marina V. Shestakova, Filip Surmont, Hirotaka Watada, Jesús Medina, Iichiro Shimomura, Gabriela Luporini Saraiva, Andrew Cooper, Antonio Nicolucci

**Affiliations:** ^1^ Department of Medicine, Diabetes Unit, Rio de Janeiro State University, Rio de Janeiro, Brazil; ^2^ Saint Luke’s Mid America Heart Institute, Kansas City, MO, United States; ^3^ Medical/Payer Evidence Statistics, BioPharmaceuticals Medical, AstraZeneca, Gaithersburg, MD, United States; ^4^ Evidera, Barcelona, Spain; ^5^ BioPharmaceuticals Medical, AstraZeneca, Cambridge, United Kingdom; ^6^ Primary Care Diabetes and Vascular Medicine, University of Leicester, Leicester, United Kingdom; ^7^ Institute for Biometrics and Epidemiology, German Diabetes Centre, Düsseldorf, Germany; ^8^ Endocrinology Research Centre, Diabetes Institute, Moscow, Russia; ^9^ BioPharmaceuticals Medical, AstraZeneca, Luton, United Kingdom; ^10^ Graduate School of Medicine, Juntendo University, Tokyo, Japan; ^11^ BioPharmaceuticals Medical, AstraZeneca, Madrid, Spain; ^12^ Graduate School of Medicine, Osaka University, Osaka, Japan; ^13^ Centre for Outcomes Research and Clinical Epidemiology, Pescara, Italy

**Keywords:** type 2 diabetes, observational study, socioeconomic factors, glycemic control, glucose-lowering drug

## Abstract

DISCOVER is a 3-year observational study program of 15,983 people with type 2 diabetes initiating second-line glucose-lowering therapy in 38 countries. We investigated the association between socioeconomic status and both the availability of a baseline glycated hemoglobin (HbA1c) measurement and poor glycemic control (HbA1c level ≥ 9.0%) in participants enrolled in DISCOVER. Factors associated with a lack of baseline HbA1c measurement or an HbA1c level ≥ 9.0% were assessed using three-level hierarchical logistic models. Overall, 19.1% of participants did not have a baseline HbA1c measurement recorded. Lower-middle country income (vs. high) and primary/no formal education (vs. university education) were independently associated with a reduced likelihood of having a baseline HbA1c measurement (odds ratio [95% confidence interval]: 0.11 [0.03–0.49] and 0.81 [0.66–0.98], respectively. Of the participants with an available HbA1c measurement, 26.9% had an HbA1c level ≥ 9.0%; 68.7% of these individuals were from lower- or upper-middle-income countries. Factors associated with an increased likelihood of poor glycemic control included low country income, treatment at a site with public and/or governmental funding (vs. private funding) and having public or no health insurance (vs. private). A substantial proportion of DISCOVER participants did not have an HbA1c measurement; more than one-quarter of these participants had poorly controlled type 2 diabetes. Both individual- and country-level socioeconomic factors are associated with the quality of care regarding glycemic control. Awareness of these factors could help improve the management of patients with type 2 diabetes.

## Introduction

Socioeconomic status is defined as a composite of an individual’s social and economic positions within a given social structure (1) and low socioeconomic status has been shown to be associated with an increased risk of developing type 2 diabetes (2, 3). Globally, type 2 diabetes is most prevalent among materially and socially deprived people, with approximately 80% of people with type 2 diabetes residing in low- or middle-income countries (4, 5).

Socioeconomic status may influence access to healthcare and quality of care, as well as a patient’s knowledge of type 2 diabetes and the resulting adherence to their disease management plans (6, 7). Studies of patients from high-income countries in Europe and North America have investigated the association between individual socioeconomic factors and health outcomes such as glycemic control (7–11). However, this relationship has been largely understudied among patients from low- and middle-income countries. Research on the socioeconomic factors associated with the management of patients with type 2 diabetes in these settings will help provide context for improving disease management in these populations.

DISCOVER is a 3-year, global, prospective, observational study program which enrolled patients from a range of lower-middle, upper-middle and high income countries. The study is designed to describe disease management patterns and a broad range of associated outcomes in people with type 2 diabetes initiating a second-line glucose-lowering treatment. Second-line therapy was defined as either adding or switching between therapies after first-line treatment with one or more oral glucose-lowering drugs (12, 13). In this analysis, we assessed the socioeconomic factors associated with having a missing baseline glycated hemoglobin (HbA1c) measurement and factors associated with poor glycemic control (defined in our analysis as having an HbA1c level ≥ 9.0%) at baseline.

## Methods

### Research Design

The methods for the DISCOVER study program have been reported in detail elsewhere (12, 13) and are briefly summarized below. The global DISCOVER study program comprises two similar, 3-year, non-interventional prospective studies conducted simultaneously in 38 countries: DISCOVER (NCT02322762) in 37 countries and J-DISCOVER (NCT02226822) in Japan (12, 13). The study protocols were approved by the appropriate clinical research ethics committees in each participating country and the relevant institutional review boards at each site (**Appendix**). The protocols comply with the Declaration of Helsinki, the International Conference on Harmonisation of Good Clinical Practice and the local regulations for clinical research. Participants were enrolled in DISCOVER from September 2014 to June 2016 and in J-DISCOVER from September 2014 to December 2015. Inclusion and exclusion criteria were kept to a minimum to reflect the diversity of patients treated in routine clinical practice (**Supplementary Table S1**). Patients with type 2 diabetes were eligible for the study if they were initiating second-line glucose-lowering therapy (defined as adding a glucose-lowering drug or switching between therapies) provided that their first-line therapy was not an injectable agent (as these individuals were likely to represent a sicker population, and therefore not be representative of the general management of patients with type 2 diabetes) or a herbal remedy/natural medicine alone. All eligible patients were invited to participate in the study by their physician and provided written informed consent. A list of participating investigators can be found in the **Appendix**.

### Data Collection

Data were collected at baseline (initiation of second-line therapy) using a standardized case report form and were transferred to a central database *via* a web-based data capture system. In line with the observational nature of the study, clinical variables such as HbA1c levels were measured and recorded in accordance with routine clinical practice; data collection was not compulsory for any of the clinical variables.

### Statistical Analysis

Participants who had data available at baseline (initiation of second-line glucose-lowering therapy) were eligible for inclusion in the present analysis. Data from China were excluded, owing to complete data being unavailable at the time of the analysis because of changes in regulatory requirements during the study. Data from Canada and France were excluded as no data were recorded for site type (primary care, general or community hospital, university or teaching hospital, specialist diabetes center or other) or ethnicity, respectively. Descriptive data are presented as numbers and percentages. For continuous variables, mean values (standard deviation) and median values (interquartile range) are reported as appropriate.

Three-level hierarchical logistic models, with participants nested in countries, were used to assess socioeconomic factors (**Supplementary Table S2**) associated with having a missing baseline HbA1c measurement and with having a baseline HbA1c level ≥ 9.0%. Patient-, site- and country-level socioeconomic factors were included in the model as fixed effects for baseline covariates (country income, site type, site location, site funding, physician specialty, insurance status, living arrangement, smoking status, sex, ethnicity, age, education level and employment status). Country income was estimated by gross national income per capita in DISCOVER countries in 2015, using the Atlas method (14), based on data from the World Bank. The Atlas conversion factor uses a country’s exchange rate for the current and preceding 2 years, adjusted for the difference between the rate of inflation in that country and international inflation. A baseline HbA1c level of 9.0% was chosen as the cut-off for poor glycemic control as this value was well above the level of 7.0% typically recommended by guidelines (15), especially for patients early on in their disease trajectory such as those initiating second-line therapy. Multiple imputation was used to account for missing data; 12.2% of participants had data missing for at least one covariate. A *P* value of < 0.05 was considered to be statistically significant.

The relative importance of the socioeconomic factors included in the multivariate analyses were estimated using the marginal *R* ^2^ value (16). Each variable was independently removed from the multivariate model and the resulting *R* ^2^ was calculated to determine the relative importance of each variable on the fit of the model. The lower the resulting *R* ^2^ value, the more important the removed variable was to the fit of the model. Imputation was carried out using IVEware (University of Michigan, MI, USA). All other statistical analyses were carried out using the SAS statistical software system (SAS Institute, Inc., Cary, NC, USA).

## Results

### Participant Disposition and Baseline Demographics

In total, 14,049 participants (87.9%) were included in the present analysis and, of these, 11,359 participants (80.9%) had baseline HbA1c levels recorded. Baseline characteristics of the overall population and of participants with or without HbA1c data are summarized in **Table 1**. Physician and site characteristics overall and by participants with or without HbA1c are summarized in **Table 2**. Most participants were either Asian (6,315/14,049, 45.1%) or Caucasian (3,898/14,049, 27.9%), with a mean age of 57.4 years (standard deviation: 12.0 years). Overall, 7,539/14,049 of participants (53.7%) were men. Participants from lower-middle-income countries accounted for a higher proportion of participants with a missing baseline HbA1c measurement (1,390/2,682, 51.8%) than of those with a recorded HbA1c measurement (3,037/11,359, 26.7%). The proportion of participants receiving combinations of metformin and a dipeptidyl peptidase-4 inhibitor as second-line therapy was higher in those with a baseline HbA1c measurement than in those without (26.7% vs. 14.7%, **Supplementary Table S3**). The opposite was observed for combinations of metformin and a sulfonylurea (19.2% vs. 32.8%, **Supplementary Table S3**).

**Table 1:** Baseline demographic and clinical characteristics of DISCOVER study participants overall, and with and without an available baseline HbA1c measurement.

Characteristic	Total (N = 14,041)	No HbA1c data available (n = 2,682)	HbA1c data available (n = 11,359)			
			HbA1c < 9.0% (n = 8,308)		HbA1c ≥ 9.0% (n = 3,051)	Overall (n = 11,359)
**Male, n (%)**	7,539 (53.7)	1,208 (45.0)	4,595 (55.3)		1,736 (56.9)	6,331 (55.7)
**Age, years, mean (SD)**	57.4 (12.0)	56.4 (11.8)	58.9 (12.0)		54.1 (11.2)	57.6 (12.0)
**Ethnicity, n (%)**						
Caucasian	3,898 (27.9)	510 (19.1)	2,597 (31.3)		791 (26.0)	3,388 (29.9)
Black	308 (2.2)	182 (6.8)	84 (1.0)		42 (1.4)	126 (1.1)
Mixed	213 (1.5)	103 (3.9)	66 (0.8)		44 (1.4)	110 (1.0)
Asian	6,315 (45.1)	1,425 (53.5)	3,660 (44.1)		1,230 (40.5)	4,890 (43.2)
Hispanic	942 (6.7)	281 (10.5)	456 (5.5)		205 (6.7)	661 (5.8)
Arabic	2,147 (15.3)	133 (5.0)	1,323 (16.0)		691 (22.7)	2,014 (17.8)
Other	172 (1.2)	31 (1.2)	105 (1.3)		36 (1.2)	141 (1.2)
Missing, n	46	17	17		12	29
**HbA1c level, %, mean (SD)**	8.3 (1.7)	–	7.5 (0.8)		10.5 (1.4)	8.3 (1.7)
Missing, n	2,682	2,682	0		0	0
**Country GNI^a^ n (%)**						
Lower-middle	4,427 (31.5)	1,390 (51.8)	1,983 (23.9)		1,054 (34.5)	3,037 (26.7)
Upper-middle	4,229 (30.1)	1,053 (39.3)	2,134 (25.7)		1,042 (34.2)	3,176 (28.0)
High	5,385 (38.4)	239 (8.9)	4,191 (50.4)		955 (31.3)	5,146 (45.3)
**Smoking status, n (%)**						
Non-smoker	9,610 (70.2)	2,175 (83.0)	5,388 (66.6)		2,047 (68.7)	7,435 (67.2)
Ex-smoker	2,243 (16.4)	262 (10.0)	1,533 (18.9)		448 (15.0)	1,981 (17.9)
Current smoker	1,837 (13.4)	182 (6.9)	1,169 (14.4)		486 (16.3)	1,655 (14.9)
Missing, n	351	63	218		70	288
**Education level, n (%)**						
No formal education	390 (3.0)	81 (3.2)	193 (2.5)		116 (4.1)	309 (2.9)
Primary (1–6 years)	1,980 (15.1)	451 (17.7)	1,012 (13.2)		517 (18.2)	1,529 (14.5)
Secondary (7–13 years)	6,487 (49.6)	1,254 (49.1)	3,909 (50.9)		1,324 (46.6)	5,233 (49.7)
University or higher (≥ 13 years)	4,215 (32.2)	766 (30.0)	2,564 (33.4)		885 (31.1)	3,449 (32.8)
Missing, n	969	130	630		209	839
**Employment status, n (%)**						
Employed	6,647 (49.1)	1,076 (41.1)	3,147 (39.4)		1,196 (40.9)	5,571 (51.0)
Not employed	3,988 (29.5)	1,045 (39.9)	2,090 (26.1)		853 (29.2)	2,943 (27.0)
Retired	2,765 (20.4)	481 (18.4)	1,868 (23.4)		416 (14.2)	2,284 (20.9)
Disabled or other	136 (1.0)	16 (0.6)	95 (1.2)		25 (0.9)	120 (1.1)
Missing, n	505	64	313		128	441
**Health insurance, n (%)**						
Private	2,014 (14.9)	283 (10.8)	1,251 (15.6)		480 (16.5)	1,731 (15.9)
Public or governmental	7,989 (59.1)	1,033 (39.3)	5,267 (65.9)		1,689 (58.2)	6,956 (63.8)
Mixed	354 (2.6)	52 (2.0)	220 (2.8)		82 (2.8)	302 (2.8)
None	3,168 (23.4)	1,261 (48.0)	1,256 (15.7)		651 (22.4)	1,907 (17.5)
Missing, n	516	53	314		149	463

Percentages were calculated for all patients with data available; patients with missing data were excluded. ^a^Countries were categorized by 2015 gross national income per capita into lower-middle-income (US$1,005–3,955), upper-middle-income (US$3,956–12,235) and high-income (≥ US$12,236) countries. GNI, gross national income; HbA1c, glycated hemoglobin; SD, standard deviation.

**Table 2:** Physician and site characteristics of DISCOVER study participants overall, and with and without an available baseline HbA1c measurement.

Characteristic	Total (N = 14,041)	No HbA1c data available (n = 2,682)	HbA1c data available (n = 11,359)		
			HbA1c < 9.0% (n = 8,308)	HbA1c ≥ 9.0% (n = 3,051)	Overall (n = 11,359)
**Site type, n (%)**					
Primary care	4,603 (32.9)	601 (22.4)	3,013 (36.4)	989 (32.5)	4,002 (35.4)
General or community hospital	1,897 (13.6)	349 (13.0)	1,163 (14.1)	385 (12.7)	1,548 (13.7)
University or teaching hospital	2,046 (20.8)	180 (6.7)	1,327 (16.0)	539 (17.7)	1,866 (16.5)
Specialist diabetes center	2,914 (20.8)	843 (31.5)	1,446 (17.5)	625 (20.5)	2,071 (18.3)
Other	2,533 (18.1)	705 (26.3)	1,324 (16.0)	504 (16.6)	1,828 (16.2)
Missing, n	48	4	35	9	44
**Site location, n (%)**					
Urban	11,763 (84.2)	2,484 (92.8)	6,599 (79.9)	2,680 (88.1)	9,279 (82.1)
Rural	2,211 (15.8)	194 (7.2)	1,655 (20.1)	362 (11.9)	2,017 (17.9)
Missing, n	67	4	54	9	63
**Site funding, n (%)**					
Public or governmental	4,389 (31.6)	575 (21.6)	2,704 (32.9)	1,110 (36.9)	3,814 (34.0)
Private	9,377 (67.5)	2,089 (78.4)	5,422 (65.9)	1,866 (62.1)	7,288 (64.9)
Mixed	133 (1.0)	1 (0.0)	103 (1.3)	29 (1.0)	132 (1.2)
Missing, n	142	17	79	46	125
**Physician specialty, n (%)**					
Primary care physician	1,957 (14.0)	547 (20.4)	1,076 (13.0)	334 (11.0)	1,410 (12.5)
Endocrinology or diabetology	9,314 (66.6)	1,758 (65.6)	5,389 (65.2)	2,167 (71.2)	7,556 (66.8)
Internal medicine	2,185 (15.6)	318 (11.9)	1,387 (16.8)	480 (15.8)	1,867 (16.5)
Other	527 (3.8)	57 (2.1)	409 (5.0)	61 (2.0)	470 (4.2)
Missing, n	58	2	47	9	56

Percentages were calculated for all patients with data available; patients with missing data were excluded. HbA1c, glycated hemoglobin.

### Factors Associated With Having a Missing Baseline HbA1c Measurement

**Figure 1:**
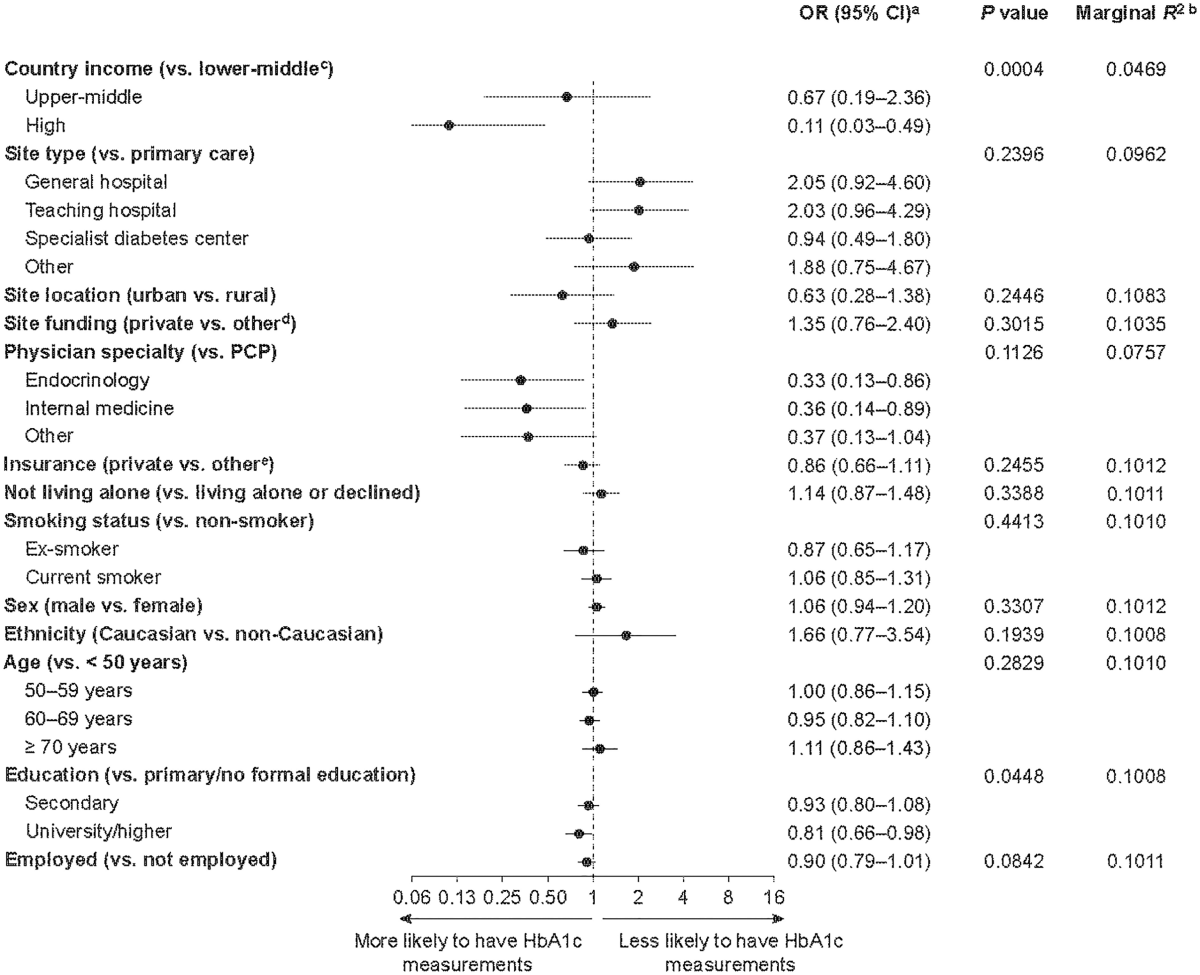
Factors associated with a lack of HbA1c measurements in patients enrolled in the DISCOVER study at baseline. ^a^ORs were calculated using three-level hierarchical models, with patients nested in sites and sites nested in countries and were adjusted for all variables in the figure. ^b^Each *R* ^2^ was calculated when the corresponding variable was removed from the model. The lower the resulting *R* ^2^ value, the more important the removed variable was to the fit of the model. ^c^Countries were categorized by 2015 gross national income per capita into lower-middle-income (US$1,005–3,955), upper-middle-income (US$3,956–12,235) and high-income (≥ US$12,236) countries. ^d^Public or governmental, or mixed, funding. ^e^Public, mixed or none. CI, confidence interval; HbA1c, glycated hemoglobin; OR, odds ratio; PCP, primary care physician.

Factors associated with a missing baseline HbA1c measurement are shown in **Figure 1**. Living in a lower-middle-income country (vs. a high-income country), being treated by a primary care practitioner (vs. an endocrinologist or internist) and having received only primary or no formal education (vs. university/higher education) were associated with an increased likelihood of a participant lacking a baseline HbA1c measurement. On the basis of both the significant *P* value (0.0004) and the low marginal *R* ^2^ value (0.047) on the removal of gross national income from the model, lower country income was identified as the most important risk factor associated with a lack of baseline HbA1c measurement.

### Factors Associated With Poor Glycemic Control at Baseline

**Figure 2:**
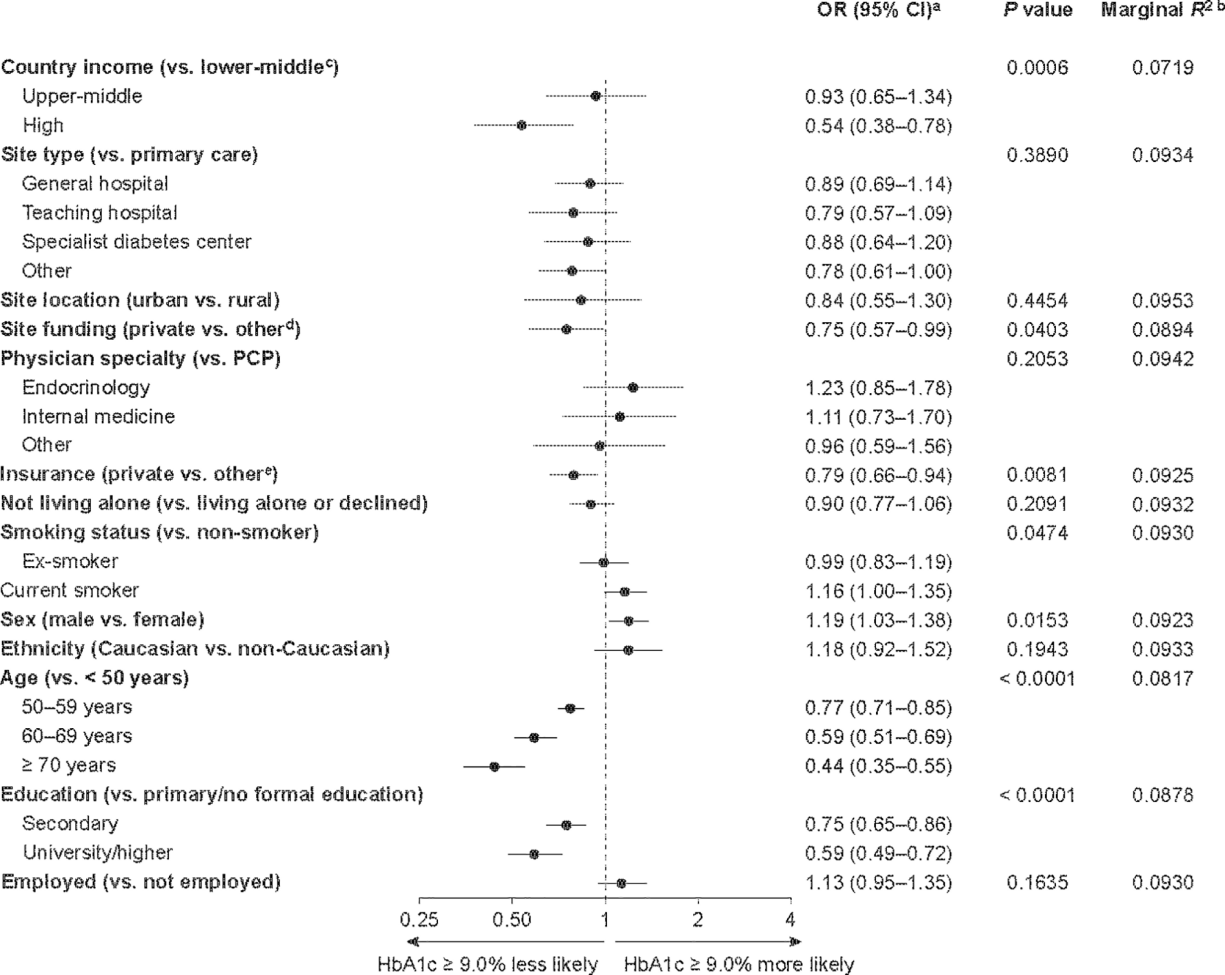
Factors associated with having an HbA1c level ≥ 9.0% among DISCOVER study patients initiating a second-line glucose-lowering therapy. ^a^ORs were calculated using three-level hierarchical models, with patients nested in sites and sites nested in countries and were adjusted for all variables in the figure. ^b^Each *R* ^2^ was calculated when the corresponding variable was removed from the model. The lower the resulting *R* ^2^ value, the more important the removed variable was to the fit of the model. ^c^Countries were categorized by 2015 gross national income per capita into lower-middle-income (US$1,005–3,955), upper-middle-income (US$3,956–12,235) and high-income (≥ US$12,236) countries. ^d^Public or governmental, or mixed, funding. ^e^Public, mixed or none. CI, confidence interval; HbA1c, glycated hemoglobin; OR, odds ratio; PCP, primary care physician.

Of the participants with an available baseline HbA1c measurement, 3,051/11,359 participants (26.9%) had an HbA1c level ≥ 9.0% (**Table 2**). Participants from lower-middle- and upper-middle-income countries accounted for a higher proportion of participants with an HbA1c level ≥ 9.0% (2,096/3,051, 68.7%) than those with an HbA1c level < 9.0% (4,117/8,308, 49.6%). Factors associated with poor glycemic control at baseline (HbA1c level ≥ 9.0%) are shown in **Figure 2**. Socioeconomic factors associated with an increased likelihood of poor glycemic control included living in a lower-middle-income country (vs. a high-income country), being treated at a site with public or governmental, or mixed, funding (vs. private funding), having public or no health insurance (vs. private) and having received primary or no formal education (vs. secondary or university/higher education). Male sex and being younger than 50 years were also associated with an increased likelihood of having an HbA1c level ≥ 9.0%. Lower-middle country income (vs. high-income), being younger than 50 years and having only primary or no formal education were identified as the three most important risk factors for having a baseline HbA1c level ≥ 9.0%, based on their low *P* values (0.0006, < 0.0001 and < 0.0001, respectively) and low marginal *R* ^2^ values (0.072, 0.082 and 0.088, respectively).

## Discussion

In this study, we have shown that several socioeconomic factors are associated with missing baseline HbA1c measurements or poor glycemic control in people with type 2 diabetes at initiation of second-line glucose-lowering therapy. Low country income was independently associated with both outcomes. This finding has important implications, given that diabetes is most prevalent in populations of people with low socioeconomic status (4). Moreover, people with type 2 diabetes and poor glycemic control are at higher risk of diabetes-related complications and incur higher healthcare costs than those with good glycemic control (17, 18).

Noticeably, almost 20% of DISCOVER participants were lacking a baseline HbA1c measurement. This is of particular concern given that participants were all initiating second-line glucose-lowering therapy at the time of study enrolment. Although fasting plasma glucose and postprandial plasma glucose measurements may have been employed at some study sites, a previous DISCOVER publication revealed that 7.1% of participants had no recorded measure of baseline blood glucose levels (19). Factors that correlated with a lack of available HbA1c data in our study included living in a lower-middle-income country, rather than a high-income country, and a low educational level. This is consistent with findings from a study in the USA, which found a lack of health insurance and low educational level to be associated with decreased odds of having an HbA1c measurement (20). Patients with university or higher education often have better health literacy than those with a primary or secondary education alone and may be more aware of the need to attend visits for HbA1c readings (21). Poor health literacy in DISCOVER participants with a low educational level may explain the association between education level and the availability of a baseline HbA1c measurement (21).

In our model, low country income was also highlighted as the most important socioeconomic factor associated with a participant not having a recorded baseline HbA1c measurement. Our findings echo those of previous studies, which report the limited availability of HbA1c testing in lower-middle-income countries (22–24). The few available test centers, located at hospitals and/or specialist care centers, were not accessible to patients from rural areas (23). Furthermore, a cross-sectional study of patients with type 2 diabetes in sub-Saharan Africa showed that limited access to HbA1c testing was a key factor associated with poor glycemic control (22). Country income is likely to be related to a large proportion of patients without a baseline HbA1c measurement receiving combinations of metformin and a sulfonylurea at second line. Sulfonylureas are typically cheaper and more readily available than other newer treatment classes such as dipeptidyl peptidase-4 inhibitors, and may therefore be the only treatment available to prescribers in lower-income countries where high-cost medications are unavailable.

We found that more than one-quarter of DISCOVER participants with an available baseline measurement had a baseline HbA1c level ≥ 9.0%. This relatively high level of apparent treatment inertia may be partially explained by the fact that participants were all initiating second-line glucose-lowering therapy.

Unsurprisingly, living in a lower-middle-income country, rather than a high-income country, and having a low education level were also associated with an increased likelihood of having an HbA1c level ≥ 9.0%. These findings are again consistent with those from other smaller studies (9, 10, 25, 26). In particular, the association between lower country income and poor glycemic control highlights the challenges in the management of patients with type 2 diabetes in Sub-Saharan Africa. Cross-sectional studies from this region, which contains many low- and lower-middle income countries, have consistently shown poor glycemic control and high levels of type 2 diabetes-related complications (27–31). In a systematic review and meta-analysis that included 51 studies, there was an inverse association between socioeconomic status and HbA1c levels in people with type 2 diabetes; factors associated with this result were social deprivation, low education level and unemployment (25). In a cross-sectional study of patients with diabetes in France, those who reported financial difficulties were more likely to have an HbA1c level ≥ 8.0% than those who were financially comfortable (10). Access to care centers, limited therapy choice and ability to adhere to treatment regimens may partially explain the association between country income and poor glycemic control in DISCOVER participants. Although it has been suggested that the association between low socioeconomic status and poor glycemic control could be mediated by the incidence of depression and healthcare avoidance in this population (7), findings from a recent study demonstrated that financial hardships were more consistently associated with poor glycemic control than other psychosocial factors (32).

Being younger than 50 years was also demonstrated to be an important risk factor for having an HbA1c level ≥ 9.0%. Younger patients, aged 18–39 years, have been shown to have a significantly higher risk of having poor glycemic control than those aged 75 years or older (26), making them particularly vulnerable to long-term complications of type 2 diabetes. Although this finding is concerning, studies in high-income countries have suggested that this association is due to a lack of engagement with treatment reviews, rather than low socioeconomic status (33). Given the low overall age of DISCOVER participants, implementing changes such as better type 2 diabetes education early in the disease trajectory may reduce the occurrence of long-term complications and health resource use.

Other factors associated with having an HbA1c level ≥ 9.0% included being treated at a site with public or governmental, or mixed, funding and having public or no health insurance. Interestingly, physician specialty was not associated with poor glycemic control, suggesting that primary care physicians and specialists are providing a similar quality of care.

Although our study showed no relationship between having a non-Caucasian ethnicity and the likelihood of having an HbA1c level ≥ 9.0%, other studies have shown a significant impact of ethnicity on glycemic control. In a nationwide observational study from the Swedish National Diabetes Register, ethnicity was a predictor of glycemic control independent of income and education (8). A cohort study of patients from the UK found that patients of south Asian or black African/Caribbean descent had a greater chance of having poor glycemic control than Caucasian patients (9). The disparity between these findings and our own may be explained by the correlation between ethnicity and country. For example, in DISCOVER, the majority of south Asian patients are from South-East Asia, meaning that any association between ethnicity and either access to HbA1c testing or poor glycemic control cannot necessarily be separated from the country-level association.

A major strength of the DISCOVER study program is the inclusion of a large and diverse participant population, as well as participants from many lower-middle- and upper-middle-income countries who have rarely or never been studied before. Therefore, findings from DISCOVER provide a comprehensive overview of diabetes management in different clinical settings globally. DISCOVER was purposely designed to assess the management of patients with type 2 diabetes through a dedicated standardized electronic case report form. Such a study would not have been possible by extracting data from existing databases, particularly in lower-income countries where the availability of databases suitable for research and/or the breadth of data collected are limited. Our findings must also be interpreted with potential limitations in mind. Given the observational nature of the study, some countries had a high proportion of missing data, which may have reduced the precision of our analysis. The degree of missing data prevented alcohol consumption from being included in the analysis. In addition, DISCOVER included many Muslim countries where alcohol consumption is not considered socially acceptable, and may therefore be under-reported. Although sites were selected to maximize diversity, it is unclear as to whether our findings fully reflect the quality of care within each country and/or region, given that sites participating in DISCOVER may place a greater focus on patient care than sites that are not involved in the study. It is also unclear whether these findings can be extrapolated to outside the participating countries.

Our study demonstrates inequality in the management of patients with type 2 diabetes between lower-middle- and high-income countries. Patient-, country- and site-level socioeconomic factors have all been demonstrated to affect the likelihood of a lack of HbA1c recording in people with type 2 diabetes initiating second-line therapy, and also the likelihood of having poor glycemic control in those who have had their HbA1c levels recorded. The strong association between being from a country with a low gross national income and having a baseline HbA1c level ≥ 9.0% indicates that country-level socioeconomic factors, in addition to individual socioeconomic factors, play a significant role in the likelihood of a patient having poor glycemic control (7). The findings from this analysis highlight the need for further exploration of the social determinants of health and for interventions to address these determinants in patients with type 2 diabetes, particularly those residing in low-income countries.

## Data Availability Statement

Data may be obtained from a third party and are not publicly available. Data underlying the findings described in this manuscript may be obtained in accordance with AstraZeneca’s data sharing policy described at https://astrazenecagrouptrials.pharmacm.com/ST/Submission/Disclosure.

## Ethics Statement

The studies involving human participants were reviewed and approved by appropriate clinical research ethics committees in each participating country and the relevant institutional review board at each site (see **Appendix**). The patients/participants provided their written informed consent to participate in this study.

## Author Contributions

MG, FT, HC, JC-R, PF, KK, WR, MS, FS, HW, JM, IS, GS, AC and AN agreed the general content of the manuscript. MG, HC and FT developed the statistical analysis plan, which was conducted by HC and FT. MG, FT, HC, JC-R, PF, KK, WR, MS, FS, HW, JM, IS, GS, AC and AN contributed to the development of the manuscript. MG, FT, HC, JC-R, PF, KK, WR, MS, FS, HW, JM, IS, GS, AC and AN approved the final version of the manuscript before its submission. An AstraZeneca team reviewed the manuscript during its development and was allowed to make suggestions. However, the final content was determined by MG, FT, HC, JC-R, PF, KK, WR, MS, FS, HW, JM, IS, GS, AC and AN. MG is the guarantor of this work. All authors contributed to the article and approved the submitted version.

## Funding

The DISCOVER study was funded by AstraZeneca. DISCOVER is a non-interventional study, and no drugs were supplied or funded. Medical Writing support for this manuscript was provided by Lucy Ambrose DPhil and Steph Macdonald PhD of Oxford PharmaGenesis, Oxford, UK, and was funded by AstraZeneca.

## Conflict of Interest

MG, KK, WR, MS, HW, IS and AN are members of the DISCOVER Scientific Committee and received support from AstraZeneca to attend DISCOVER planning and update meetings. HC, PF, FS, JM, GS and AC are employees of AstraZeneca. JC-R is an employee of Evidera. FT is an employee of Saint Luke’s Mid America Heart Institute, which has received funding from AstraZeneca. In addition, MG has received honoraria from Merck-Serono; KK has received honoraria from AstraZeneca, Boehringer Ingelheim, Eli Lilly, Janssen, Merck Sharp & Dohme, Novartis, Novo Nordisk, Sanofi, Takeda, Servier and Pfizer, research support from AstraZeneca, Boehringer Ingelheim, Eli Lilly, Merck Sharp & Dohme, Novartis, Novo Nordisk, Sanofi and Pfizer, and also acknowledges support from the National Institute for Health Research Applied Research Collaboration–East Midlands and the Leicester Biomedical Research Centre; WR has received research support from Novo Nordisk; MS has received honoraria from AstraZeneca, Boehringer Ingelheim, Eli Lilly, Merck Sharpe & Dohme, Novo Nordisk, Sanofi and Servier, and research support from Novo Nordisk, Sanofi and Servier; HW has received honoraria from Astellas Pharma, AstraZeneca, Boehringer Ingelheim, Daiichi Sankyo, Sumitomo Dainippon Pharma, Eli Lilly, Kissei Pharmaceutical, Kowa Pharmaceuticals America Inc., Kyowa Hakko Kirin, Merck Sharp & Dohme, Mitsubishi Tanabe Pharma, Novartis, Novo Nordisk, Ono Pharmaceutical, Sanofi, Sanwa Kagaku Kenkyusho and Takeda, and research support from Abbott, Astellas Pharma, AstraZeneca, Bayer, Benefit One Health Care, Boehringer Ingelheim, Bristol-Myers Squibb, Daiichi Sankyo, Dainippon Sumitomo Pharma, Eli Lilly, Johnson & Johnson, Kissei Pharmaceutical, Kowa Pharmaceuticals America Inc., Kyowa Hakko Kirin, Merck Sharp & Dohme, Mitsubishi Tanabe Pharma, Mochida Pharmaceutical, Nitto Boseki, Novartis, Novo Nordisk, Ono Pharmaceutical, Pfizer, Sanofi, Sanwa Kagaku Kenkyusho, Taisho Toyama Pharmaceutical, Takeda and Terumo Corp; IS has received honoraria from Astellas Pharma, AstraZeneca, Boehringer Ingelheim, Kowa Pharmaceuticals America Inc., Merck Sharp & Dohme, Mitsubishi Tanabe Pharma, Novo Nordisk, Ono Pharmaceutical, Sanwa Kagaku Kenkyusho and Takeda, and research support from Astellas Pharma, AstraZeneca, Daiichi Sankyo, Eli Lilly, Japan Foundation for Applied Enzymology, Japan Science and Technology Agency, Kowa Pharmaceuticals America Inc., Kyowa Hakko Kirin, Midori Health Management Centre, Mitsubishi Tanabe Pharma, Novo Nordisk, Ono Pharmaceutical, Sanofi, Suzuken Memorial Foundation and Takeda; AN has received honoraria from AstraZeneca, Eli Lilly, Medtronic and Novo Nordisk, and research support from Artsana, Dexcom, Novo Nordisk and Sanofi.

Authors HC, PF, FS, JM, GLS and AC were employed by the company AstraZeneca. FT is an employee of Saint Luke’s Mid America Heart Institute, which has received funding from AstraZeneca.The authors declare that this study received funding from AstraZeneca. The funder had the following involvement with the study: AstraZeneca designed the DISCOVER study with input and guidance from DISCOVER Scientific Committee members. An AstraZeneca team reviewed this manuscript for scientific accuracy during its development and was allowed to make suggestions. However, the final content, analysis and interpretation of the data was determined by the authors. DISCOVER is a non‑interventional study, and no drugs were supplied or funded. Statistical analyses were conducted by the Mid America Heart Institute, Kansas City, MO, USA, and were funded by AstraZeneca. Medical writing support for this manuscript was provided by Lucy Ambrose DPhil and Steph Macdonald PhD of Oxford PharmaGenesis, Oxford, UK, and was funded by AstraZeneca.

## Publisher’s Note

All claims expressed in this article are solely those of the authors and do not necessarily represent those of their affiliated organizations, or those of the publisher, the editors and the reviewers. Any product that may be evaluated in this article, or claim that may be made by its manufacturer, is not guaranteed or endorsed by the publisher.
