# Supplementary material for: Socioeconomic Factors Associated With Glycemic Measurement and Poor HbA1c Control in People With Type 2 Diabetes: The Global DISCOVER Study
Source: Front Endocrinol (Lausanne). 2022 Apr 22;13:831676. doi: 10.3389/fendo.2022.831676 (PMC9072655; doi:10.3389/fendo.2022.831676)
Supplement: Supplementary file 1 [file DataSheet_1.docx]

**Appendix**

**Country-level clinical research ethics committees and institutional review boards involved in the DISCOVER study program**

| **Country** | **Authority/Committee Name** |
| --- | --- |
| Algeria | Central ethics committee of CHU Beni Messous |
| Argentina | Comite de Bioetica del Instituto de Investigaciones Clinicas Rosario  Comite de Bioetica en Investigacion de Ciencias de la Salud  Comite de Docencia e Investigacion, Hospital San Martin de Parana  Comite de Etica Dr Carlos Barclay  Comité de Ética y Revisión Institucional Centro Médico Famyl  Comité Independiente de Ética de Investigación en Salud Prof. Dr. Marcelino Rusculleda  Comite Provincial de Bioetica - Ministerio de Salud Provincia de Santa Fe  IEC - Comité Independiente de Ética para Ensayos en Farmacología Clínica (FEFyM)  SIPROSA Sistema Provincial de Salud |
| Australia | Bellberry Human Research Ethics Committees |
| Austria | BASG - Bundesamt für Sicherheit im Gesundheitswesen (Austria)  Ethikkommission der Medizinischen Universität Innsbruck |
| Bahrain | Research and Research Ethics Committee  Royal Medical Services, Bahrain Defence Force |
| Brazil | CEP da Faculdade de Jaguariúna (CEP-FAJ)  CEP da Faculdade de Medicina da Universidade de São Paulo - FMUSP/SP  CEP da Faculdade de Medicina de São José do Rio Preto – FAMERP  CEP da Faculdade de Medicina do ABC/SP  CEP da Irmandade da Santa Casa de Misericórdia de Porto Alegre / RS  CEP da Irmandade da Santa Casa de Misericórdia de São Paulo/ISCMSP  CEP da Pontificia Universidade Catolica de Campinas / PUC Campinas  CEP da Pontifícia Universidade Católica do Paraná - PUC/PR  CEP da Real Benemérita Associaçao Portuguesa de Beneficência - Hospital São Joaquim  CEP da Universidade Caxias do Sul / RS  CEP da Universidade Positivo  CEP do Centro Universitário de Brasília – UNICEUB  CEP do Centro Universitario Franciscano  CEP do Hospital das Clínicas da Universidade Federal de Goiás  CEP do Hospital de Clinicas da Universidade Federal do Parana - HCUFPR / PR  CEP do Hospital e Maternidade Angelina Caron/PR  CEP do Hospital Lifecenter  CEP do Hospital Moinhos de Vento/ RS  CEP do Hospital Pró-Cardíaco/RJ  CEP do Hospital Universitario - CAS/UFJF/MG  CEP do Hospital Universitário Pedro Ernesto  CEP do Hospital Universitario Walter Cantidio - HUWC / CE  CEP do Instituto de Ciência e Tecnologia - UNESP Campus de São José dos Campos  CEP Investiga - Instituto de Pesquisas  CONEP - Comissão Nacional de Ética em Pesquisa |
| Canada | Advarra’s CIRBI |
| Colombia | Comité de Bioética en Investigación Clínica Farallones  Comité de Estudios Medicos S.A.S CREIMED  Comité de Ética e Investigación Biomédica de la Fundación Valle del Lili  Comité de Ética en Investigación Clínica de la Costa  Comite de Etica en Investigacion del Centro Medico Imbanaco  Comité de Ética en Investigación. Medplus Medicina Prepagada S.A.  Comite de Etica en la Investigacion CAIMED  Comite de Investigaciones y Etica en Investigaciones Hospital Pablo Tobon Uribe  Comite Institucional de Etica e Investigacion Clinica  Comité Institucional de Ética en Investigación C.I.E.I CAFAM |
| Costa Rica | Comité Ético Científico del Instituto Costarricense de Investigaciones Clínicas  Comité Ético Cientifico Universidad Ciencias Médicas |
| Czech Republic | Státní ústav pro kontrolu léčiv |
| Denmark | No ethics committee approval was needed for the DISCOVER study |
| Egypt | Ministry of Health & Population Central Directorate for Research and Health Development |
| France | CPP Sud Ouest et Outre Mer II |
| India | Clinical Trial Ethics Committee  CLINICOM  Ethics Committee - Apollo Hospitals  Ethics Committee of Diabetes Thyroid Hormone Research Institute  Ethics Committee, Diacon Hospital (Diabetes Care and Research Center)  Ethics Committee, Inamdar Multispecialty Hospital  Independent Ethics Committee, BYL Nair Hospital & TN Medical College  Institutional Ethics Committee, BGS Global Hospital  Institutional Ethics Committee Clinical Studies  Institutional Ethics Committee Fortis Hospital  Institutional Ethics Committee of Jothydev's Diabetics and Research centre  Institutional Ethics Committee of Kovai Diabetes Speciality Centre and Hospital  Institutional Ethics Committee of Rama Krishna Mission Seva Prathisthan  Institutional Ethics Committee, Apollo Gleneagles Hospitals  Institutional Ethics Committee, Oyster and Pearl Hospital  Institutional Ethics Committee, Poona Medical Research Foundation pune  Institutional Ethics Committee-The Calcutta Medical Research Institute  Integrity Ethics Committee  League Health Ethics Committee  Manipal University Ethics Committee  Medanta Institutional Ethics Committee  Medisys clinisearch ethical review board  St John’s Medical College & Hospital Institutional Ethics Committee  The Ethics Committee, Dr. V. Seshiah Diabetes Research Institute, Dr. Balaji Diabetes Care Centre  Virtuous Institutional Medical Research Ethics Committee |
| Indonesia | Ethical Committee of Research Faculty of Medicine Brawijaya  Komisi Etik Penelitian Fakultas Kedokteran Universitas Udayana/RSUP Sanglah Denpas  The Committee of Medical Research Ethics of The Faculty of Medicine University of Indonesia |
| Italy | AIFA - Agenzia Italiana del Farmaco  CE MI Area 3  Comiitato Etico Unico (CEUR) per la Basilicata  Comitato Etico Area Vasta Nord Ovest  Comitato Etico ASST Sette Laghi  Comitato Etico dell’Area Vasta Emilia Nord (AVEN)  Comitato Etico della Azienda Ospedaliera Ospedale Treviglio-Caravaggio di Treviglio  Comitato Etico della Romagna  Comitato Etico dell'Azienda Ospedaliero-Universitaria Ospedali Riuniti di Foggia  Comitato Etico dell'IRCCS Multimedica di Sesto San Giovanni  Comitato Etico Lazio 2  Comitato Etico Milano Area 3  Comitato Etico Referente per l'area di Pavia |
| Jordan | Institutional Review Board Jordan University of Science and Technology King Abdulla University Hospital International Pharmaceutical Research Center Istishari Hospital institutional review board Jordan University Hospital institutional review board Royal medical services institutional review board |
| Kuwait | Research Affairs/Ethical Review Committee, Dasman Diabetes Institute |
| Lebanon | American University of Beirut institutional review board  Ethics committee of Mount Lebanon Hospital  Hammoud hospital institutional review board  Institutional review board Rafik Hariri University hospital |
| Malaysia | Human Research Ethics Committee USM (HREC)  Medical Research & Ethics Committee  National Medical Ethics Comittee |
| Mexico | COFEPRIS  Comision de Bioetica del Instituto Nacional de Cardiologia Ignacio Chavez  Comite de Etica de la Fac de Med de la UANL y Hospital Universitario "Dr. Jose Eleuterio Gonzalez"  Comite de Etica del Instituto Jalisciense de Investigacion Clinica  Comite de etica en Investigacion de Investigacion Biomedica para el Desarrollo de Farmacos  Comite de Etica en Investigacion de la Unidad de Investigacion en Salud de Chihuahua, S.C.  Comité de Ética en Investigación de México Centre for Clinical Research S.A. de C.V.  Comite de Etica en Investigacion de Unidad Clinica de Bioequivalencia S. de R.L. de C.V.  Comité de Ética en Investigación del Centro de Estudios de Investigación Metabólicos y Cardio. S.C.  Comite de Etica en Investigacion del Centro Hospitalario Vicor, SA de C.V. CHG Hospitales  Comite de Etica en Investigacion del Hospital General de Mexico  Comite de Etica en Investigacion del INCMNSZ (Nutricion)  Comite de Etica en Investigacion del Instituto del Corazon de Queretaro SA de CV  Comite de Etica en Investigacion del Instituto Jalisciense de Investigacion Clinica, S.A. de C.V. |
| Netherlands | METC St Elisabeth |
| Norway | Regional Committees for Medical and Health Research Ethics |
| Oman | Ethics & Scientific Research Committee and Royal Hospital |
| Panama | Comite de Bioetica de la Investigacion del Instituto Conmemorativo Gorgas de Estudios de la Salud |
| Poland | Komisja Bioetyczna przy OIL w Katowicach |
| Russian Federation | EC at First Clinical Emergency Hospital n.a. E.E.Volosevish  EC at Saint-Petersburg SBHI “City hospital №40 of Resort District”  EC under SEIHPE Novosibirsk State Medical University  Independent Interdisciplinary Committee for Ethical Examination of Clinical Studies  Independent Interdisciplinary Committee for Ethical Examination of Clinical Studies  LEC at FGBU Endocrinology Research Center of Minzdrav of Russia  LEC at FSAEI HE " First Moscow State Medical University n.a. I.M. Sechenov" of the MoH of the RF  LEC at FSBSI “Scientific Research Institute of Therapy and Preventive Medicine”  LEC at LLC "Best Clinical Practice"  LEC at NIH "Central Clinical Hospital # 2 n.a.N.A. Semashko of OJSC "Russian Railways"  LEC at SBEI HPE "Moscow State Medical and Dentistry University n.a. A. I. Evdokimov" of the MoH  LEC at SBHI «City Clinical Hospital #10 of Kanavinsky District»  LEC at SHI of Yaroslavl region “Clinical Hospital №2”  LEC to City Cinical Hospital named after F.I. Inozemtseva  Reg. Endocrinol. Dispensary  SBIH of Moscow "Endocrinilogy Dispensary" of Department of Healthcare of Moscow  SEIHPE "Rostov SMU of MoH of RF"  SPB SHI "Pokrovskaya City Hospital"  The Independent Multidisciplinary Committee on Ethical Review of Clinical Trials |
| Saudi Arabia | KFMC IRB (King Fahad Medical City)  Khamis Mushayt Armed Forces Hospital Research Ethics Committee  KSUMC-IRB (King Saud University Medical city) IMC-IRB (International Medical Center) SGH-IRB (Saudi German Hospital) |
| Spain | Agencia Española del Medicamento y Productos Sanitarios (Spain)  CEIC Autonomico de Andalucia  CEIC del Area de Segovia  CEIC del Hospital Universitario de Fuenlabrada  CEIC Fundació Gol i Gurina  CEIC Hospital Clínico de Salamanca  CEIC Hospital Uiversitario Virgen del Rocio  CEIC Hospital Universitario Central de Asturias  CEIC Hospital Universitario de Elche  CEIC Hospital Universitario Puerta del Mar  CEIC Hospital Universitario Ramon y Cajal  CEIC Hospital Universitario Virgen Macarena  CEIC Hospital Virgen de la Arrixaca  Fundacion Jimenez Diaz  Hospital Universitari i Politecnic La Fe  Hospital Universitario de La Princesa  RA Asturias  RA Castilla y Leon  RA Cataluna  RA Comunidad Valenciana  RA Madrid  RA Murcia |
| South Africa | Pharma Ethics Research Ethics Committee  Stellenbosch University Health Research Ethics Committee  University of Cape Town Research Ethics Committee |
| South  Korea | IRB of Bundang Jesaeng hospital  IRB of Chung-Ang University Hospital  IRB of Eulji University Hospital  IRB of Gangnam Severance Hospital, Yonsei University Health System  IRB of Gwangmyung Seongae hospital  IRB of Hyewon Medical Foundation SeJong General Hospital  IRB of Jeju National University Hospital  IRB of Kangwon National University Hospital  IRB of Myongji Hospital  IRB of Seoul National University Bundang Hospital  IRB of Severance Hospital, Yonsei University Health System  IRB of SoonChunHyang University Hospital Cheonan  IRB of Wonkwang University Hospital |
| Sweden | Regionala etikprövningsnämnden i Stockholm |
| Taiwan, Republic of China | Chang Gung Medical Foundation, Institutional Review Board  Changhua Christian Hospital Institutional Review Board  Cheng Hsin General Hospital, Institutional Review Board  Chi Mei Medical Center, Institutional Review Board  Chia-Yi Christian Hospital Institutional Review Board  China Medical University Hospital, Institutional Review Board  Chung Shan Medical University Hospital, Institutional Review Board  Institutional Review Board of the Cathay General Hospital  Mackay Memorial Hospital, Institutional Review Board  National Cheng Kung University Hospital, Institutional Review Board  National Taiwan University Hospital, Research Ethics Committee  Research Ethics Review Committee Far Estern Memorial Hospital  Taichung Veterans General Hospital, Institutional Review Board  Taipei Veterans General Hospital, Institutional Review Board  Tri-Service General Hospital, Institutional Review Board |
| Tunisia | Comité d’éthique de l’Institut National de Nutrition de Tunis (LEC)  Comité de Protection des Personnes Nord de l'institut Pasteur de Tunis (CPP-N) (CEC) |
| Turkey | Erciyes University Medical Faculty Ethics committee |
| United Arab Emirates | Dubai Health Authority  IRB Sheikh Khalifa Medical City |

**Investigators participating in the DISCOVER study program**

Kahlifa Abdallah,

Talaat Abdelaaty,

Alaa Abdelsalam,

Azidah Abdul Kadir,

Ashraf Abgad,

Roberto Abrão Raduan,

Manal Abu Shady,

Koussay Ach,

Yehya Adonis,

Alina Agafyina,

Megha Rani Agrawal,

Carlos Aguilar Salinas,

Noor Shahrazat Ahmad Hamad,

Sven Ahnlund,

Diego Aizenberg,

Yasmeen Ajaz,

Barış Akıncı,

Mounira Al Arouj,

Noor Al Busaidi,

Mwafag Al Hyari,

Dhekra Al Naqeeb,

Awad Al Qahtani,

Fayez Al Ruawilly,

Mohamed Al Shareef,

Mahmoud Al sheikh,

Norsiah Ali,

Insaf Ali,

Gustav Allerstrand, Mireille Amm,

Fawaz Ammari,

A Amod,

Nadia Amor Jemel,

Kjell Andersson,

Lars Andersson,

Fernando Andres,

Nicoline Angergård,

Firas Annabi,

Mikhail Antsiferov,

Mustafa Araz,

Maria Arechavaleta Granell,

Marcelo Arruda Nakazone,

Ralph Audehm,

Awaitef Awwad ,

Samir Azar,

Malha Azzouz,

Anna Carla Babini,

Hajer Ballout,

Karin Barbosa Kersten Moraes,

Christina Barklund,

Maria Barreda Gonzalez,

Dagmar Bartaskova,

Osman Başpınar,

Fahri Bayrm,

Krim Belkacem,

Mohamed Ben Lassoued,

Leila Ben Salem,

Ferroudja Benbouazza,

Abdullah Bennakhi,

Lars Benson

Vinay Bhat,

Arpandev Bhattacharya,

Sujit Bhattacharya,

Jon Erik Billington,

Albert Boada Valmaseda,

Irina Bondar,

Abderrahmene Bouazdi,

Samia Bourezane,

Fatma Boussema,

Helmut Brath,

Marilia Brito Gomes,

Mansour Brouri,

Ole Petter Brunstad,

A.J.S.H. Brussen,

Peter Bety,

Yavuz Beyazıt,

Ruifang Bu,

L Burgess,

Rune Burkeland-Matre,

Mehmet Çalan,

Gloria Cánovas Molina,

Sille Capio,

Nacu Aureo Caracas Portilla,

Luz Ángela Casas,

Antonio Ceriello,

Bong Soo Cha,

Chwen-Tzuei Chang,

Nadia Charfi,

Tirthankar,

Rosa Collazos,

Victor Commendatore,

Francesc Xavier Cos Claramunt,

João Lindolfo Cunha Borges,

Eugenia Czajkowska-Kaczmarek,

Wu Dai,

Paola D'Angelo,

Ferdinandus de Looze,

Maristela de Oliveira Beck,

R.J. De Vos,

Eva Decroli,

Gary Chaudhury,

Alexendre Cheaib,

Roger Chen,

Hui Chen,

Jung-Fu Chen,

Chih Hao Chen Ku,

Elena Chernaya,

Anwer Cherrak,

Alejandro Chertkoff,

Chung Gu Cho,

Sung Hee Choi,

Giuseppe Citro,

Alessandra Ciucci,

Alejandro Cob Sanchez,

K Coetzee,

Ramis Çolak Deed,

Giuseppe Derosa,

Suneel Deshpande,

Graziano Di Cianni,

Tonatiu Diaz Escalante,

Barbora Diepoltova,

Guillermo Dieuzeide,

H.A. Dirkse,

Michiel Dogger,

Nicholas Doong,

Rahul Dosi,

Ana Clarissa Duin Fortes,

Irina Dvoryashina,

Grzegorz Dzida,

Christoph Ebenbichler,

Akram Echtay,

Tomas Edelsberger,

Stig Ekkert,

Salah El Ghazaly,

Abdelaziz El Kady,

Nabil El Kafrawy,

Manal El Masry,

Lubna El toni,

Kamil El Zein,

Yasser Elganiny,

Hassan Elian,

Freddy Eliaschewitz,

Peter Elmegaard,

Gamal Elnaggar,

Ahmad Elomari,

Ramzi Eltarazi,

Javier Elvira Gonzalez,

Yulia Ermakova,

Diaa Ewais,

Mohamed Fahmy Abdelaziz,

Banah Faisal,

Tem Lom Fam,

Ihab Fardon,

Nabil Fawzy,

Anika Fenando,

Felipe Ferre Larrosa,

Pablo Fletcher,

Evelyn Fliesser-Görzer,

Martin Forseth,

Fadlo Fraige Filho,

Gustavo Daniel Frechtel,

Mario Fritsch Neves,

Graciela Viviana Fuente,

M Fulat,

Geirmund Furnes,

Kristian Furuset, Ahmed

Gahzzawi, Samir Gamil,

S Ganesh, Mounir Garali,

Pedro Alberto Garcia Hernandez,

Luis Garcia Ortiz,

Elizabeth Gelersztein,

Bruno Geloneze Neto,

Kim Gerlöv,

Yehia Ghanem,

Ottavio Giampietro,

Peter Giles,

Jorge Archibaldo Glenny,

Milton Moyses Golbert,

Fernando Gomez Peralta,

Alexander Gonzalez Dorado,

Guillermo Gonzalez Galvez,

Amelia Gonzalez Gamarra,

Juan Jose Gorgojo Martinez,

Bozena Gornikiewicz-Brzezicka,

Bettina Gregersen,

Kirsten Grete Walaas,

M Greyling,

Maria del Pilar Grimaldo de Sucre,

Mingjun Gu,

Andrea Gudrun Berz,

Jugal Gupta,

Sanjay Gupta,

Sonsoles Gutiérrez Medina,

Suzae Hachim,

Jihad Haddad,

Khadija Hafidh,

Adlane Hallal,

Ivar Halvorsen,

Jassin Hamed,

Ursula Hanusch,

Chris Harrison,

Edith Hartmann,

Abdulhameed Hassan,

Peter Hay,

Eric Hernandez Triana,

Mohamed Hesham El Hefnawy,

John Hickey,

Kristin Hjörleifsdottir,

Joost Hoekstra,

Hans Högström,

Frits Holleman,

Stian Holmvik,

Bertram Hölzl, F Hoosen,

Petra Horanska,

Jakub Hron,

Ming-Chia Hsieh,

Ji Hu,

Chien-Ning Huang,

Yi-Jen Hung,

Chii-Min Hwu,

Jaime Orlando Ibarra Gomez,

Attia Ibrahim,

Ahmed Ismail abdelghany,

Rina Istarowati,

Kaveh Izadkhasti,

Hana Jachymova,

Per Ola Jahr,

Kamel Jallouli,

Monica Jaramillo Sanchez,

Said Jebril,

Dennis Jersewski,

Guangshan Ji,

Linong Ji,

Ju-Ying Jiang,

Carola Johansson,

Catharina Jonason,

Siti Juhariah,

Sandeep Julka,

Ferdinandus Kakiay,

Shailaja Kale,

Sanjay Kalra,

Michael Kaltoft,

Shaheer Kamal,

Mesbah Kamel,

Rafic Kannan,

Sülbiye Karaburgu,

Igor Karen,

Alexandra Kautzky-Willer,

Ghassan Kawar,

Mark Kennedy,

Jose Francisco Kerr Saraiva,

Jothydev Kesavadas,

Hesham Khalfan,

Mustapha Kharroubi,

Mohamed Khattab,

Oleg Khmelnitskyi,

Alexander Khokhlov,

Reza Khusrawi,

Chong Hwa Kim,

JaeTaek Kim,

SangJin Kim,

SangWook Kim,

Yong-Hyun Kim,

Herlin Kisworini,

Anna Klein,

German Henry

Kleisinger,

A Kok,

Fei Ping Kow,

Nils Kragh,

Elena Krasilnikova,

Zdenka Krejsova,

Ove Kristianslund,

Ida Ayu Kshanti,

Yaşar Küçükardalı,

Ferit Kerim Küçükler,

Weimar Kunz Sebba

Barroso de Souzaem,

Milan Kvapil,

Yuanita A. Langi,

Estêvão Lanna Figueiredo,

Daan Lansdorp,

Johan Larsson,

Nagwa Lasheen,

Norbert Lazuka,

Jae Hyuk Lee,

Sang Ah Lee,

Chun-Chuan Lee,

Aniceto Leguizamo Dimas,

Minxiang Lei,

Stephen Leow,

Dorota Lesniewska-Krynska,

Qifu Li,

Xuefeng Li,

Yiming Li,

Yufeng Li,

Yukun Li,

Shaoda Lin,

Ching-Ling Lin,

Anders Lindh,

G.A. Lochorn,

Kristin Løland Jacobsen,

Claudio Esteban Lopez,

Bassem Louzir,

Song Lu,

Bernhard Ludvik,

Dilcia Maria Lujan,

Jianhua Ma,

Noureddine Maalem,

Zeina Maani,

Josef Machacek,

Laura Maffei,

Renan Magalhães Montenegro Junior,

Hisham Magd,

Mohan N. Magdum,

AA Mahomed,

Debashish Maji,

H Makan,

Rachid Malek,

Emilia Malicherova,

Joanna Malicka,

Alexey Malikov,

Miguel Angel Mangas Cruz,

Paolo Marenco,

Mats Martinell,

Mohamed Mashaheet, Stanislaw Mazur,

Marie Mehreb,

Tatiana Meleshkevich,

Juan Francisco Merino Torres,

H.G.M. Mevissen,

Noel Mikha,

E Mitha,

Ambrish Mithal,

Ashot Mkrtumyan,

Zhaohui Mo,

Rihab Mohamed El Nour,

Fatiha Mohammedi,

Norhaliza Mohd Ali,

Rudy Mokodompit,

Dora Inés Molina de Salazar,

Britta Mollerup,

Munther Momani,

Cristobal Morales Portillo,

Mohsen Mostafa Khaled,

Mikael Mullaart,

Luciana Muniz Pechmann,

Svetlana Mustafina,

Yvan Mwana,

Tejaswi N,

Mazen Naba,

Mary Nabil,

Rudolf Najjar,

Thomas Nathow,

Hisham Nazer,

Edgar Nessim,

Luis Alejandro Nevarez Ruiz,

Jana Nevrla,

N Ngcakani,

Nik Harlina Roza Nik Kazim,

Sudzilla Nordin,

Youesef Obeid,

Ashraf Okba,

Atilla Önmez,

H Oosthuizen,

Horng-Yih Ou,

Haroun Ouertani,

Mohamed Ouertani,

Ufuk Özuğuz,

T Padayachee,

Francisco Gerardo Padilla Padilla,

Tianrong Pan,

Daniel Panarotto,

Jong Suk Park,

Kangseo Park,

Seok O Park,

Bernhard Paulweber,

Emilia Pelayo Orozco,

Vasil Pema,

Márcio Antônio Pereira,

Hikmat Permana,

Nina Petunina,

Teresa Cristina Piscitelli Bonansea,

Grazyna Popenda,

Petr Potuznik,

Rakesh Prasad,

Dalton Precoma,

Jiri Pumprla,

Shen Qu,

Virginia Laureana Quevedo Martin,

Lea Raclavska,

Amine Rahou,

Sheetal Raj M,

Yuli Rakhmawati,

Balamurugan Ramanathan,

Luis Carlos Ramirez,

Xingwu Ran,

N Ranjith,

Srinivasa Rao,

J Reddy,

Jannike Reymer,

José Augusto Ribas Fortes,

David Richmond,

Ciro Manuel Riojas Charles,

Lucas Rista,

Javier Riveiro,

Giselle Madeline Rodriguez Mendez,

Luis Gerardo Rodriguez Torres,

Daoud Roula,

Eman Roushdy,

Khaled Rubean,

Ahmed Saad Salama,

Mohamed Saad Zaghloul,

Sherif Sabry,

Yasser Saed,

Samir Saha,

Mounzer Saleh,

Jesus Sanchez Pardo,

Luis Sanchez Vadillo,

Dhiãnah Santini de Oliveira Chachamovitz,

Made Ratna Saraswati,

Ramazan Sarı, Laksmi Sasiarini,

Ahmed Sayed Badawy,

Gerit Schernthaner,

Richard Seiberlich,

Gaetano Serviddio,

Habib Sfar,

Essam Shabaan,

Dilip Shah,

Inas Shaltout,

Paramesh Shamanna,

Mazen Sharairi,

Jonathan Shaw,

Ehsan Sheikhan,

Salah Shelbaya,

Eman Shesha,

Marina Shestakova,

Wayne H-H Sheu,

Lixin Shi, Roy Panusunan Sibarani,

K. P. Singh,

Ewa Skokowska,

Claudio Slon,

Djoko Wahono Soeatmadji,

B.H.F. Sombekke,

Neale Somes,

Weihong Song,

Bhavana Sosale,

Radhakrishna Sothiratnam,

Harald Sourij,

Jaganmani Sreekanth,

Andrzej Stankiewicz,

Andre Storm-Larsen,

Finn Strekerud,

Krzysztof Strojek,

Rishi Sukla,

Tri Sutowo,

Lars Henrik T Sutterud,

Ireneusz Szymczyk,

Sri Wahyu Taher,

Hussein Tayyib,

Karim Temimi,

Anders Tengblad,

Tamer Tetiker,

Sten Thorslund,

Tadeusz Tomala,

Şenay Topsakal,

Ivonne Aimee Torres Quiroz,

Shih-Tzer Tsai,

Rahul Tulle,

Rene Turcinek,

Zinet Turki Marrakchi,

Alparslan Kemal Tuzcu,

Kader Uğur,

Irina Ulyanova,

Galina Varvarina,

Esdras Martin Vasquez,

ZFA Vawda,

Kjersti Veholmen,

Paranthaman Vengadasalam,

Ida Veng-Christenssen,

S Venkataraman,

Alica Vesela,

Maria Helena Vidotti,

Maricela Vidrio Velazquez,

Balaji Vijayam,

Juan Villagordoa Mesa,

Adriana Alicia Villarino,

María Fernanda Villegas Otalora,

Mary Vinocour Fornieri,

Rafael Violante Ortiz,

Sergey Vorobyev,

Saria Wakim,

Thomas Wascher,

Norbert Watzinger,

Charlotte Weirsøe,

M.G.J. Willink,

Mirella Wojtecka-Grabka,

Alan Wright,

Bin Wu,

Guangda Xiang,

Zaiton Yahaya,

Sunjie Yan,

Tao Yang,

Wei-Shiung Yang,

Yin Yee Kian,

Feng-Chieh Yen,

Müge Özsan Yılmaz,

Xuefeng Yu,

Ebtisam Zakaria,

Rozita Zakaria,

Ivana Zavaroni,

Jitka Zemanova,

Lihui Zhang,

Ying Zhang,

Jun Zhu,

Mei Zhu,

Hendra Zufry.
