# Supplementary material for: Socioeconomic Factors Associated With Glycemic Measurement and Poor HbA1c Control in People With Type 2 Diabetes: The Global DISCOVER Study
Source: Front Endocrinol (Lausanne). 2022 Apr 22;13:831676. doi: 10.3389/fendo.2022.831676 (PMC9072655; doi:10.3389/fendo.2022.831676)
Supplement: Supplementary file 2 [file DataSheet_2.docx]

**Supplementary Table S1.** Inclusion and exclusion criteria of the DISCOVER study program.

| **Inclusion criteria** |
| --- |
| - Diagnosis of type 2 diabetes - Age ≥ 18 years^a^ - Initiating a second-line therapy (add-on or switching) after a first-line oral treatment with monotherapy, dual therapy, or triple therapy^b^ - Provision of written informed consent |
| **Exclusion criteria** |
| - Type 1 diabetes - Pregnancy - Initiation of dual therapy after having previously received two different lines of monotherapy (e.g. initiation of a combination of a sulfonylurea and a dipeptidyl peptidase-4 inhibitor after successive metformin monotherapy and sulfonylurea monotherapy) - Current treatment with chemotherapy or oral or intravenous steroids - Undergoing dialysis or has had a renal transplant - First-line treatment was insulin or other injectable agent^c^ - First-line treatment was herbal remedies/natural medicines alone - Participation in an interventional trial - Condition/circumstance, which, in the opinion of the investigator, could significantly compromise the 3-year follow-up (e.g. life-threatening comorbidities, tourist, non-native speaker, or does not understand the local language where interpreter services are not reliably available, psychiatric disturbances, dementia, alcohol or drug abuse) - Not willing to sign the informed consent form |

^a^In Japan, age ≥ 20 years. ^b^In Japan, only patients using an oral monotherapy as first-line treatment were included. ^c^Patients who received short-term initial treatment with insulin followed by oral therapy were eligible if the treatment with insulin lasted no more than 2 weeks and occurred at least 6 months before initiation of second-line therapy. In such patients, insulin was considered not as a first-line treatment, but as an acute treatment to lower glycemic levels quickly before starting regular treatment.

**Supplementary Table S2.** Socioeconomic and demographic factors of DISCOVER study patients assessed in the present analysis.

| **Socioeconomic factors** | **Other factors** |
| --- | --- |
| • Country gross national income (according to the 2015 World Bank classification)^a^  • Site type (primary care, general or community hospital, university or teaching hospital, specialist diabetes center or other)  • Site location (urban or rural)  • Site funding (private or other)  • Physician specialty (primary care practitioner, endocrinology, internal medicine or other)  • Health insurance coverage (private, public or mixed)  • Living situation (living alone or not living alone)  • Smoking status (non-smoker, ex-smoker or current smoker)  • Education level (primary, secondary, university or higher or no formal education)  • Employment status (employed or not employed) | • Sex (male or female)  • Ethnicity (Caucasian or non-Caucasian)  • Age (<50 years, 50–59 years, 60–69 years or ≥ 70 years) |

^a^Countries were categorized by 2015 gross national income per capita into lower-middle-income (US$1,005–3,955), upper-middle-income (US$3,956–12,235) and high-income (≥ US$12,236) countries. Countries were as follows: lower-middle income (Egypt, India, Indonesia, Jordan and Tunisia); upper-middle income (Algeria, Argentina, Brazil, Colombia, Costa Rica, Lebanon, Malaysia, Mexico, Russia, South Africa and Turkey); and high income (Australia, Austria, Bahrain, Canada, Czech Republic, Denmark, France, Italy, Japan, Kuwait, Netherlands, Norway, Oman, Panama, Poland, Saudi Arabia, South Korea, Spain, Sweden, Taiwan and United Arab Emirates).

**Supplementary Table S3.** Second-line treatment regimens of DISCOVER study participants overall, and with and without an available baseline HbA1c measurement.

| **Second-line treatment, n (%)** | **Total**  **(N = 14,041)** | **No HbA1c data available**  **(n = 2,682)** | **HbA1c data available (n = 11,359)** | | |
| --- | --- | --- | --- | --- | --- |
|  |  |  | **HbA1c  < 9.0% (n = 8,308)** | **HbA1c  ≥ 9.0% (n = 3,051)** | **Overall  (n = 11,359)** |
| Metformin monotherapy | 245 (1.7) | 62 (2.3) | 162 (2.0) | 21 (0.7) | 183 (1.6) |
| Sulfonylurea monotherapy | 349 (2.5) | 71 (2.6) | 207 (2.5) | 71 (2.3) | 278 (2.4) |
| DPP-4 inhibitor monotherapy | 583 (4.2) | 93 (3.5) | 447 (5.4) | 43 (1.4) | 490 (4.3) |
| Other monotherapy^a^ | 496 (3.5) | 179 (6.7) | 255 (3.1) | 62 (2.0) | 317 (2.8) |
| Metformin + sulfonylurea | 3,058 (21.8) | 881 (32.8) | 1,509 (18.2) | 668 (21.9) | 2,177 (19.2) |
| Metformin + DPP-4 inhibitor | 3,429 (24.4) | 394 (14.7) | 2,504 (30.1) | 531 (17.4) | 3,035 (26.7) |
| Metformin + other^a^ | 2,689 (19.2) | 537 (20.0) | 1,555 (18.7) | 597 (19.6) | 2,152 (18.9) |
| Other combinations^a^ | 2,331 (16.6) | 292 (10.9) | 1,435 (17.3) | 604 (19.8) | 2,039 (18.0) |
| Insulin^b^ | 860 (6.1) | 173 (6.5) | 233 (2.8) | 454 (14.9) | 687 (6.0) |

Percentages were calculated for all patients with data available; patients with missing data were excluded. ^a^Excluding insulin. ^b^On its own or as part of combinations. DPP-4, dipeptidyl peptidase-4; HbA1c, glycated hemoglobin.
